# Unleashing the TLR9-driven multilineage differentiation of myeloid leukemia cells *in vivo*

**DOI:** 10.1016/j.omtn.2024.102430

**Published:** 2025-01-02

**Authors:** Katarzyna Piwocka

**Affiliations:** 1 Nencki Institute of Experimental Biology, Polish Academy of Sciences, Warsaw, Poland

## Main text

Evading the physiological recognition and destruction by the immune system is one of the emerging hallmarks of cancer. In acute myeloid leukemia (AML), a heterogeneous malignancy with a high incidence of relapse, a central feature is the blockade of cellular differentiation at an early stage and defective response to differentiation stimuli. This leads to the accumulation of immature cells unrecognized by the immune system and, finally, to expansion and disease development.^1^^,^^2^ Such resistance to effective immune response and differentiation occurs even if innate immune receptors such as Toll-like receptor 9 (TLR9) (CpG oligonucleotide) are highly expressed.

AML shows the accumulation of immature myeloid cells and immune evasion based on intrinsic mechanisms.^3^ Although immunotherapeutic strategies have shown promising results in a number of hematological malignances, their effectiveness and positive clinical outcomes in AML are limited, partly due to immune evasion.^4^^,^^5^ Therefore, there is an urgent need to develop novel concepts and strategies to unleash the immune response to open the door for effective immunotherapies in AML. Recent discoveries have focused attention on the signal transducer and activator of transcription 3 (STAT3), which is predominantly recognized as a proleukemic prosurvival transcription factor and a driver of survival, proliferation, angiogenesis, and metastasis.^6^ However, its role as a master regulator of immune evasion has only been revealed in the last few years.^7^ It was noted by the Kortylewski team that activated STAT3 also promotes the expression of immunosuppressive factors while inhibiting T helper 1 (Th1) immunostimulatory molecules, thus playing a critical role in ensuring AML immune evasion.^8^ Therefore, it is not surprising that the possibility of reprogramming and unblocking differentiation and inhibiting STAT3 raised interest as a potentially effective therapy. However, many previous studies showed that the effectivity of STAT3 inhibition by pharmacological drugs was very limited due to a lack of enzymatic activity, or that strategies based on antisense and decoy molecules were not effective if they lacked targeted delivery.^9^^,^^10^

In the July 2024 issue of the *Molecular Therapy Nucleic Acids*, Wang et al. described a decoy oligonucleotide-based strategy to unblock multilineage differentiation of acute myeloid leukemic cells, activation of T cell-mediated immune response, and regression of acute myeloid leukemia in mice.^11^ To address this issue, authors used bi-functional TLR9-targeted decoy oligodeoxynucleotide (CpG-STAT3d), which inhibited STAT3 specifically in TLR-9^+^ target cells. The concept was based on a previous demonstration by Kortylewski and colleagues that eliminating STAT3 signaling in leukemia using small interfering RNA or decoy DNA strategies permits TLR9-induced AML cell differentiation and thus leads to potent adaptive immune responses and leukemia regression.^12^^,^^13^^,^^14^^,^^15^^,^^16^

Wang and colleagues demonstrated that such a strategy leading to the inhibition of STAT3 and and/or DNA methyltransferase 1 (DNMT1) epigenetic control mediates the TLR-9-driven reprogramming of leukemic cells. The findings show the immunotherapeutic potential of the TLR9-driven therapy and suggest that therapies based on bi-functional oligonucleotides have broader relevance to several AML subtypes, and possibly also to other malignancies. The studies revealed that the CpG-STAT3d used in humanized and mouse models significantly reduced engraftment and the development of leukemia. Limited leukemia cell engraftment in the spleen and bone marrow was associated with strong infiltration by macrophages, neutrophils, and CD8^+^ T cells, together with elevated markers of Th1 immune response. Additionally, significant expansion of leukemic cells expressing myeloid differentiation markers (CD11b/MHC-II/CD86) was observed. Importantly, such a response was not visible when only CpG was applied without STAT3 simultaneous inhibition. Wang and colleagues also showed that using the combined TLR9-immunostimulation and STAT3 inhibition in a syngeneic model harboring CBFB/MYH11 gene fusion (which enables T cell recognition), led to CD4^+^ and CD8^+^ T cell-mediated leukemia eradication and long-term animal survival.

More specifically, the authors found that a combination of TLR9 activation with STAT3 inhibition by CpG-STAT3d results in upregulated myeloid differentiation associated with the elevated expression of myeloid markers, antigen presentation-related genes, and interferon (IFN) responses, as well as a reduction in *Runx*, a leukemia-promoting transcription factor. The following single-cell transcriptomic analysis (scRNA sequencing) revealed that CpG-STAT3d induced multilineage differentiation of AML cells into immune-activated subsets such as monocytes/macrophages, erythroblasts, and B cells. Importantly, CpG-STAT3d-treated animals showed a decline in the proliferating AML cells associated with an expansion of the potentially immunogenic cell subsets with the IFN-related and antigen-presenting functions.

Finally, Wang et al. discovered a novel role for STAT3 in maintaining AML epigenetic stability, leading to a blockade of myeloid differentiation and immunogenicity. Wingelhofer et al. found that the differentiation of leukemic myeloid cells is regulated on the epigenetic level by the activity of the DNA methyltransferase DNMT1.^17^ Furthermore, STAT3 regulates the expression of and interacts with DNMT1. As inhibition of DNMTs is involved in the TLR9-driven leukemia cell differentiation, the treatment with CpG-STAT3d resulted in the downregulation of methyltransferases and the upregulation of myeloid differentiation-related genes. Accordingly, the combination of DNMT inhibition using azacitidine with CpG oligonucleotides alone mimicked CpG-STAT3d effects. This resulted in AML cell differentiation, T cell activation, and systemic leukemia regression. Importantly, effective targeting and inhibition of STAT3 in leukemic cells together with TLR9 stimulation led to epigenetic remodeling, myeloid differentiation, and elimination of malignant cells by the T cell immune response.

### Conclusions

Defective cellular differentiation is a critical feature of AML. This also has been recognized recently as a major player, and hence it became a target of many currently investigated novel therapies. With a significant interest in linking the TLR-driven differentiation to therapy in AML and other leukemias,^18^^,^^19^ as well as epigenetic therapies remodeling differentiation, elements regulating differentiation are now under investigation in early clinical trials. Thus, targeting the regulatory mechanisms involved in myeloid differentiation by using the efficient biological systems may provide an attractive way to improve therapeutic outcomes in AML. In summary, Wang et al. identified an efficient and highly promising strategy based on targeted bi-functional CpG-STAT3d, which led to AML cell reprogramming and myeloid differentiation and resulted in the activation of T cells and AML regression *in vivo*. This highlights the immunotherapeutic potential of this TLR9-driven therapy to lead to concurrent STAT3 and/or DNMT inhibition. In a more general context, the proposed strategy based on bi-functional oligonucleotides paves the way for further studies aimed at other AML subtypes and possibly also other malignancies.

## Acknowledgments

This work was supported by National Science Centre grant 2021/41/B/NZ5/04077 to K.P.

## Declaration of interests

The author declares no competing interests.
